# Trajectories of Posttraumatic Stress in Youths After Natural Disasters

**DOI:** 10.1001/jamanetworkopen.2020.36682

**Published:** 2021-02-15

**Authors:** Betty S. Lai, Annette M. La Greca, Ahnalee Brincks, Courtney A. Colgan, Michelle P. D’Amico, Sarah Lowe, Mary Lou Kelley

**Affiliations:** 1 Department of Counseling, Developmental, and Educational Psychology, Lynch School of Education and Human Development, Boston College, Chestnut Hill, Massachusetts; 2 Department of Psychology, University of Miami, Miami, Florida; 3 Department of Epidemiology and Biostatistics, Michigan State University, East Lansing; 4 School of Public Health, Georgia State University, Atlanta; 5 Department of Social and Behavioral Sciences, Yale School of Public Health, New Haven, Connecticut; 6 Department of Psychology, Louisiana State University, Baton Rouge

## Abstract

**Importance:**

Disaster exposure is associated with the development of posttraumatic stress (PTS) symptoms in youths. However, little is known about how to predict which youths will develop chronic PTS symptoms after disaster exposure.

**Objective:**

To evaluate PTS symptom trajectories among youths after 4 major US hurricanes and assess factors associated with those trajectories.

**Design, Setting, and Participants:**

This cohort study used integrative data analysis to combine data from 4 studies of youths’ responses to natural disasters (hurricanes Andrew [1992], Charley [2004], Ike [2005], and Katrina [2008]) at time points ranging from 3 to 26 months after the disasters. Those studies recruited and surveyed youths aged 6 to 16 years at schools via convenience sampling of schools near the path of destruction for each hurricane. This study was conducted from August 2017 to August 2020, and pooled data were analyzed from February 2019 to October 2020.

**Exposure:**

Experience of a natural disaster during the ages of 6 to 16 years.

**Main Outcomes and Measures:**

Posttraumatic stress symptoms were assessed using the University of California, Los Angeles, Posttraumatic Stress Disorder Reaction Index (UCLA PTSD-RI) and the UCLA PTSD-RI-Revised. Latent class growth analyses were used to evaluate the youths’ PTS symptom trajectories and associated factors.

**Results:**

Among 1707 youths included in the study, the mean (SD) age was 9.61 (1.60) years, 922 (54%) were female, and 785 (46%) self-identified as White non-Hispanic. Four PTS symptom trajectories were identified: chronic (171 participants [10%]), recovery (393 [23%]), moderate-stable (563 [33%]), and low-decreasing (580 [34%]). Older youths were less likely to be in the chronic group; compared with the chronic group, each 1-year increase in age was associated with increased odds of being in the other groups (recovery: odds ratio [OR], 1.78 [95% CI, 1.29-2.48]; moderate-stable: OR, 1.94 [95% CI, 1.43-2.62]; and low-decreasing: OR, 2.71 [95% CI, 1.99-3.71]). Compared with males, females had higher odds of being in the chronic group than in any other group (recovery group: OR, 0.48 [95% CI, 0.26-0.91]; moderate-stable group: OR, 0.37 [95% CI, 0.21-0.64]; and low-decreasing group: OR, 0.25 [95% CI, 0.14-0.44]).

**Conclusions and Relevance:**

In this cohort study, few youths reported chronic distress, and trajectories among most youths reflected recovery or low-decreasing PTS symptoms. Older age and identification as male were factors associated with decreased odds of a chronic trajectory. Youths with chronic or moderate-stable trajectories may benefit from intervention.

## Key Points

**Question:**

What are the trajectories of posttraumatic stress (PTS) symptoms among youths after natural disasters, and what factors are associated with those trajectories?

**Findings:**

This cohort study of 1707 US youths exposed to major hurricanes identified 4 PTS symptom trajectories: chronic (10%), recovery (23%), moderate-stable (33%), and low-decreasing (34%). Female and younger youths experienced more severe PTS symptom trajectories.

**Meaning:**

The findings suggest that a substantial number of youths may experience chronic or moderate-stable PTS symptom trajectories after a natural disaster and might benefit from intervention.

## Teaser

This cohort study uses integrated data from 4 studies of US youths exposed to major hurricanes to assess trajectories of posttraumatic stress symptoms and investigate factors associated with those trajectories.

## Introduction

Natural disasters are associated with the mental health of children. Approximately 100 million youths globally are exposed to disasters each year.^1,2^ After disasters, primary presenting psychological symptoms among youths are posttraumatic stress (PTS) symptoms.^3,4,5^ Elevated rates of PTS symptoms among youths are as high as 72% during the first 3 months after a disaster.^6^ In the long term, PTS symptoms among youths are associated with poorer mental and physical health, academic performance, and vocational outcomes.^7,8,9,10,11^ Thus, it is important to understand and address postdisaster PTS symptoms among youths.

Stepped care models are current best practice in addressing youths’ postdisaster PTS symptoms.^12,13^ Stepped care models triage youths based on assessments of PTS symptoms and other forms of psychological distress. After assessment, only youths at highest risk for chronic distress after a disaster should receive the most intensive interventions, whereas those at lowest risk receive supportive care. This stepped care approach is necessary because of the large number of youths affected by disasters alongside the limited funding available for mental health and social services after disasters.^14,15^

However, a barrier to implementing postdisaster stepped care models is a lack of clarity regarding how to triage youths based on risk for persistent PTS symptoms. This barrier exists because it is unclear how youths’ initial PTS symptom presentations after disasters are associated with their long-term PTS symptoms. Few studies have assessed youths at multiple time points after a disaster, and even fewer studies have assessed them beyond the first year after a disaster.^16,17^ In addition, researchers have primarily assumed that all youths in disaster-affected areas follow a similar pattern of response to a disaster. However, abundant evidence from the literature on adults^18,19^ and emerging evidence from the literature on children^20,21^ indicates that people have different long-term patterns, or trajectories, of psychological responses to traumatic events such as disasters.

Among adults, robust evidence exists for 4 prototypical trajectories of PTS symptoms after a disaster.^22,23^ Across studies, these trajectories are typically labeled *chronic*, characterized by high levels of persistent PTS symptoms that do not remit over time; *recovery*, characterized by initially elevated PTS symptoms followed by a decrease in symptoms over time; *resilient*, characterized by low levels of PTS symptoms over time; and *delayed*, characterized by elevated levels of PTS symptoms that emerge more than 6 months after the disaster event. However, adult research provides only limited insight into youths’ postdisaster responses because the experiences of youths before and after a disaster are distinct from those of adults.^24,25,26^

Researchers have begun to examine youths’ PTS symptom trajectories following traumatic events, but findings are inconsistent.^27,28,29^ Therefore, it is unclear what typical PTS symptom trajectories occur among youths after disasters. Studies on youths’ PTS symptom trajectories differ with regard to the trajectories identified and the proportion of youths in each trajectory.^27,28,29^ Variability in existing studies on youths’ PTS symptom trajectories makes it difficult to interpret, compare, and resolve discrepant findings. Discrepant findings may be associated with differences in the disaster event examined, assessment timing, the analysis type, sample recruitment, or risk factors examined.

One way to provide more robust information on prototypical trajectories of PTS symptoms among youths exposed to disasters is to use integrative data analysis, which allows researchers to combine participant information from individual data sets into 1 large data set by statistically harmonizing data so that all data may be analyzed jointly. Integrative data analysis allows researchers to apply consistent analyses (eg, latent class growth analysis) to the data to obtain more robust estimates of PTS symptom trajectories and the proportion of youths experiencing each PTS symptom trajectory type.

With use of integrative data analysis and latent class growth analysis, this study pooled data from studies of 4 of the most destructive hurricanes in US history: hurricanes Andrew, Charley, Ike, and Katrina. Hurricanes are important to study because the frequency and intensity of severe storms are projected to increase owing to climate change.^30^ The objectives of this study were to assess trajectories of youths’ PTS symptoms beyond the first year after a disaster and to evaluate factors associated with those trajectories. On the basis of the literature,^31,32,33,34^ we expected to observe 3 to 4 trajectories of PTS symptoms; we also expected participants who were younger, female, or racial/ethnic minorities to be classified in more-distressed trajectories.^35,36,37,38^ Of note, youths of racial/ethnic minority groups are more likely to experience racism and social contexts that are associated with greater risk of harm during disasters.^35^

## Methods

This cohort study pooled data from 4 studies of psychological distress among youths after disasters: hurricanes Andrew (1992), Charley (2004), Katrina (2005), and Ike (2008).^20,34,37,39^ Participants in those studies included 1707 youths aged 6 to 16 years who were recruited from elementary, middle, and high schools in Texas, Louisiana, and Florida that were located in or near each hurricane’s path of destruction (Andrew, 568 youths; Charley, 384; Katrina, 426; and Ike, 329) (Table 1). All youths were administered paper-and-pencil surveys in a group setting, with research assistants in the room to facilitate and answer questions. The time between hurricane landfall and baseline data collection for each of the 4 studies ranged between 3 and 9 months (Table 1). The present study was conducted from August 2017 to August 2020. The pooled data were analyzed from February 2019 to October 2020. The Georgia State University and Boston College institutional review boards deemed this study exempt from institutional review board approval and informed consent because it involved secondary data analysis only. This study followed the Strengthening the Reporting of Observational Studies in Epidemiology (STROBE) reporting guideline.

**Table 1.** Posttraumatic Stress Symptoms Among Youths at Postdisaster Assessment Time Points in Studies Included in the Integrated Data Analysis

Disaster	Age range, y	Youths meeting elevated posttraumatic stress symptom cutoffs by time after the disaster, No. (%)										
		3 mo	5 mo	7 mo	8 mo	9 mo	10 mo	14.5 mo	15 mo	20.5 mo	21 mo	26 mo
Hurricane Andrew^20^^,^^a^	7-12	203/568 (35.7)	NA	109/514 (21.2)	NA	NA	72/458 (15.7)	NA	NA	NA	NA	NA
Hurricane Charley^37^^,^^b^	6-13	NA	NA	NA	NA	50/384 (13.0)	NA	NA	NA	NA	23/245 (9.4)	NA
Hurricane Ike^39^^,^^b^	7-12	NA	NA	NA	72/329 (21.9)	NA	NA	NA	37/329 (11.2)	NA	NA	NA
Hurricane Katrina^34^^,^^b^	8-16	NA	41/388 (10.8)	NA	NA	NA	NA	23/367 (6.3)	NA	17/348 (4.9)	NA	13/133 (10)

Abbreviation: NA, not applicable.

^a^ Elevated distress corresponds to University of California, Los Angeles, Posttraumatic Stress Disorder Reaction Index (UCLA PTSD-RI) scores of 40 or higher on a scale of 0 to 80.

^b^ Elevated distress corresponds to UCLA PTSD-RI–Revised scores of 38 or higher on a scale of 0 to 68.

### Measures

#### PTS Symptoms

Symptoms of PTS in each of the 4 studies were assessed using 2 versions of the same measure: the University of California, Los Angeles, Posttraumatic Stress Disorder Reaction Index (UCLA PTSD-RI) and the UCLA PTSD-RI-Revised (UCLA PTSD-RI-R).^40,41^ The former assesses PTSD according to the *Diagnostic and Statistical Manual of Mental Disorders* (Third Edition Revised) diagnostic criteria, and the latter assesses symptoms based on the *Diagnostic and Statistical Manual of Mental Disorders* (Fourth Edition) criteria for PTSD. In all versions of the UCLA PTSD-RI, Likert-scale questions assess PTS symptoms, with responses ranging from “none of the time” (0) to “most of the time” (4). Both versions of the RI have previously been shown to have high test–retest reliabilities, scoring 0.94 and 0.84, respectively.^6,42^

Data collected for Hurricane Andrew used the older, 20-question version of the UCLA PTSD-RI.^38^ Participants were asked to respond to items such as “I have arguments or physical fights” and “I am afraid that the hurricane will happen again.” Total possible scores ranged from 0 to 80, with higher scores indicating higher levels of distress symptoms.^38^ Clinical cutoffs for the PTSD symptom distress scores were as follows: doubtful (0-11), mild (12-24), moderate (25-39), severe (40-49), and very severe (60-80).^43^ This version of the UCLA PTSD-RI also had cutoffs for clinically relevant PTSD or elevated distress, which corresponded to the severe or very severe categories (ie, a score of 40 or higher). Internal consistency for this measure in the Hurricane Andrew sample was high (Cronbach α = .89).^43^

Data collected for hurricanes Charley, Ike, and Katrina used the newer, 20-question UCLA PTSD-RI-R, which assessed 17 symptoms of PTSD.^34,37,39^ Total possible scores ranged from 0 to 68, with higher scores indicating a higher level of distress symptoms. Clinical cutoffs for the PTSD symptom distress scores were as follows: doubtful (0-10), mild (11-22), moderate (23-37), severe (38-53), and very severe (54-68).^39^ The UCLA PTSD-RI-R considers a score of 38 or higher as an indication of clinically relevant PTSD or elevated distress, which corresponds to the severe or very severe PTSD clinical cutoffs. Internal consistency in the pooled studies ranged from Cronbach α = 0.83 to 0.91.^4,37^ A truncated summary score, ranging from 0 to 40, was created for the purposes of the integrated data analysis and contained the 10 items common to both versions of the UCLA PTSD-RI.

#### Factors Associated With Trajectories

The participants’ demographic characteristics (ie, age, gender, and race/ethnicity) were self-reported at baseline in each of the 4 studies. Race/ethnicity was assessed in this study because it is associated with PTS symptoms.^37,38,44^ Age was coded in years. Female gender and racial/ethnic minority status were dummy coded, with female gender and racial/ethnic minority group status (ie, identifying as Black, Asian, Hispanic, Native American, or other) as the identified groups, respectively. Hurricane exposure was measured with the Hurricane-Related Traumatic Experiences questionnaire in the Hurricane Andrew study.^38^ Six yes-or-no questions measured exposure to actual life-threatening events (eg, windows or doors breaking). Items endorsed were summed, with possible scores ranging from 0 to 6.

### Statistical Analysis

Time buckets, operationalized as dummy codes (ie, TIME03 = 1 or 0), were generated to indicate whether each participant was present for data collection at a given time point. Determination of time points for each disaster was based on documentation associated with each original disaster study. This resulted in 11 time buckets: 3, 5, 7, 8, 9, 10, 14.5, 15, 20.5, 21, and 26 months after a disaster.

Descriptive analyses were conducted using SAS, version 9.4 (SAS Institute Inc).^45^ Trajectory analyses were conducted using Mplus statistical software, version 8.1 (Muthén & Muthén).^46^ Linear latent class growth analyses were conducted using the 11 time buckets. To identify the model that best represented the data, we examined the Akaike information criterion, bayesian information criterion, and sample-size–adjusted bayesian information criterion (lower values indicate greater fit); the entropy and mean classification probability range (0 to 1; higher values indicate greater accuracy); and the Lo-Mendell-Rubin adjusted likelihood ratio test. We also examined the size of the smallest trajectory class, the substantive meaning of trajectories within each model, the fit to theory, and parsimony.^47,48^ Trajectory analyses were conducted with the truncated PTS symptom scale. As a sensitivity analysis, results were replicated with full summary PTSD-RI-R scores from hurricanes Charley, Ike, and Katrina. All models adjusted latent class assignment for each hurricane study to control for between-study heterogeneity. The 3-step procedure in Mplus was used to evaluate and control for age, gender, and racial/ethnic minority status.^49^ Prevalence of symptoms among youths, mean scores, and ranges were calculated for the measures in the study. Exploratory analyses evaluated exposure as a covariate in the postestimation regressions. Significance was set at 2-tailed *P* < .05.

## Results

A total of 1707 children were included in the 4 studies. The mean (SD) age was 9.61 (1.60) years, 922 (54%) were female, and 785 (46%) self-identified as White non-Hispanic, 495 (29%) as Black non-Hispanic, 307 (18%) as White Hispanic, 68 (4%) as mixed or other race, 51 (3%) as Asian, and 1 (1%) as Black Hispanic.

**Figure 1.**
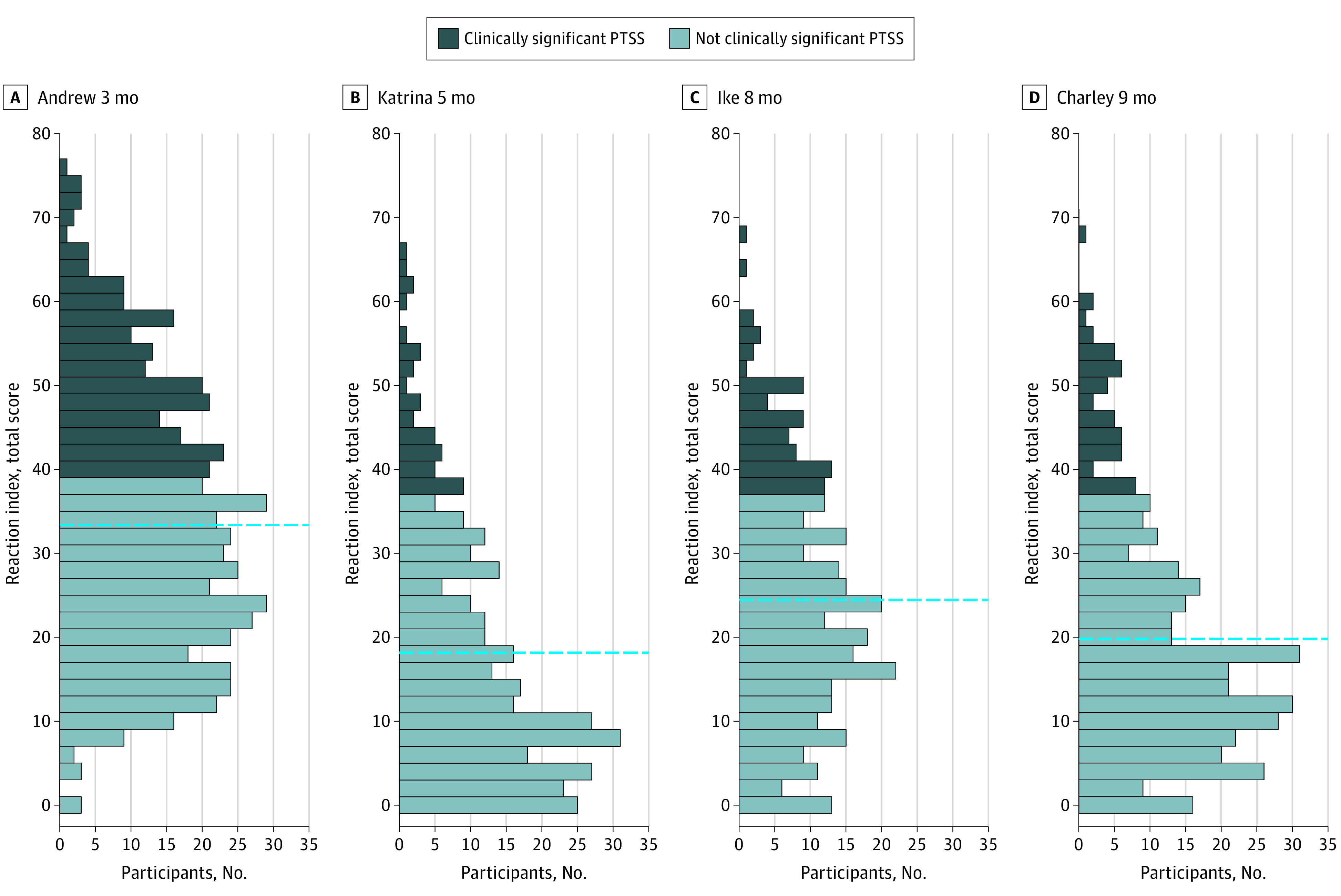
Distribution of Baseline Posttraumatic Stress Symptom (PTSS) Scores for Each Disaster Study For Hurricane Andrew, total possible scores ranged from 0 to 80, with higher scores indicating a worse symptom complex. The clinically significant cutoff was 40. For hurricanes Charley, Katrina, and Ike, total scores ranged from 0 to 68, with higher scores indicating a worse symptom complex. The clinically significant cutoff was 38. Labels indicate time after the hurricane. The dashed lines represent individual study mean PTSS.

 The prevalence of clinically relevant PTS symptoms (ie, defined as meeting the severe or very severe PTS symptom score cutoffs) among youths in this study ranged from 35.7% at 3 months after a disaster to 9.8% at 26 months after a disaster (Table 1). Figure 1 shows the distribution of baseline PTS symptoms for each disaster study.

**Figure 2.**
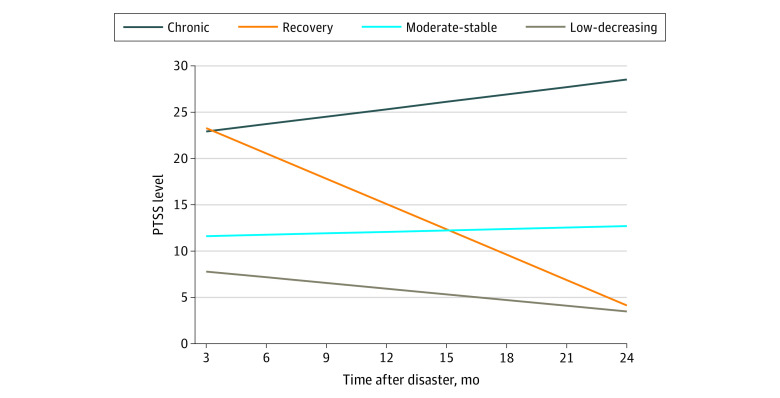
Estimated Posttraumatic Stress Symptom (PTSS) Trajectories by Latent Class

Fit indices for trajectory results are presented in Table 2. When comparing the 4- and 5-trajectory models, the proportion of the sample in 3 of the trajectories remained stable, suggesting that the 5-trajectory model was parsing the remaining trajectory into 2. Evaluating fit, theory, and existing evidence, we chose the 4-trajectory model as the final model (Figure 2). The chronic trajectory was the smallest group (171 youths [10%]), with high PTS symptom scores that increased over time (intercept [SE], 22.91 [1.34]; *P* < .001; slope [SE], 0.27 [0.10]; *P* = .006). The recovery group (393 youths [23%]) reported initially high symptom scores, but the scores decreased over time (intercept [SE], 23.28 [0.80]; *P* < .001; slope [SE], –0.91 [0.05]; *P* < .001). The moderate-stable group (563 youths [33%]) reported a moderate initial symptom scores that did not significantly change over time (intercept [SE], 11.62 [1.00]; *P* < .001; slope [SE], 0.05 [0.03]; *P* = .12). The low-decreasing group was the largest (580 youths [34%]). This group reported low symptom scores at baseline, and the scores significantly decreased over time (intercept [SE], 7.80 [0.98]; *P* < .001; slope [SE], –0.21 [0.04]; *P* < .001).

**Table 2.** Results of Latent Class Growth Models

Trajectory group, No.	AIC	BIC	Sample size–adjusted BIC	Entropy	Classification probability range	*P* value for LMR-LRT	Individuals in the smallest class, No. (%) (N = 1707)
1	28 385.82	28 472.74	28 421.91	1	1	NA	NA
2	28 161.64	28 270.30	28 206.76	0.70	0.74-0.96	<.001	373 (22)
3	28 041.43	28 182.69	28 100.09	0.68	0.65-0.94	<.001	282 (17)
4	27 949.71	28 123.57	28 021.91	0.63	0.70-0.88	.23	174 (10)
5	27 894.89	28 101.34	27 980.62	0.64	0.50-0.86	.01	129 (8)

Abbreviations: AIC, Akaike information criterion; BIC, bayesian information criterion; LMR-LRT, Lo-Mendell-Rubin adjusted likelihood ratio test; NA, not applicable in single-group models.

Latent class membership was significantly associated with the specific disaster. Compared with exposure to Hurricane Katrina, exposure to Hurricane Charley was associated with significantly higher odds of being in the chronic vs the recovery group (odds ratio [OR], 5.31; 95% CI, 1.54-18.27) and in the moderate-stable vs the recovery group (OR, 4.04; 95% CI, 1.29-12.64). Compared with exposure to Hurricane Katrina, exposure to Hurricane Ike was associated with significantly lower odds of being in the low-decreasing vs the recovery group (OR, 0.22; 95% CI, 0.11-0.42).

Older youths were less likely to be in the chronic group. Compared with the chronic group, for each 1-year increase in age, the odds of being in the recovery group increased by a factor of 1.78 (OR, 1.78; 95% CI, 1.29-2.48); the odds of being in the moderate-stable group increased by a factor of 1.94 (OR, 1.94; 95% CI, 1.43-2.62); and the odds of being in the low-decreasing group increased by a factor of 2.71 (OR, 2.71; 95% CI, 1.99-3.71). In our evaluation of gender with the chronic group as the referent outcome group, compared with males, females were more likely to be in the chronic group than in any of the other 3 trajectory groups (recovery group: OR, 0.48 [95% CI, 0.26-0.91]; moderate-stable group: OR, 0.37 [95% CI, 0.21-0.64]; and low-decreasing group: OR, 0.25 [95% CI, 0.14- 0.44]). Compared with the moderate-stable group, racial/ethnic minority youths had higher odds than nonminority youths of being in the low-decreasing group (OR, 1.91; 95% CI, 1.28-2.85) and the recovery group (OR, 2.09; 95% CI, 1.28-3.41).

Exploratory analyses suggested that higher levels of exposure to disaster were associated with greater odds of membership in the low-decreasing and recovery trajectory groups compared with the moderate-stable group. A 1-unit increase in the exposure variable was associated with an increase by a factor of 1.59 in the odds of membership in the low-decreasing trajectory group (OR, 1.59; 95% CI, 1.19-2.12) and by a factor of 2.11 in the odds of membership in the recovery group (OR, 2.11; 95% CI, 1.58-2.81) compared with membership in the moderate-stable group.

## Discussion

This cohort study found diverse responses among youths after exposure to a disaster. Within each disaster study,^20,34,37,39^ rates of clinically relevant PTS symptoms decreased over time. However, in the pooled sample, youths exhibited 4 PTS symptom trajectories: chronic, recovery, moderate-stable, and low-decreasing. When we examined factors associated with these trajectories (ie, age, gender, and racial/ethnic minority status), older age was associated with being in trajectories of less severe PTS symptoms, whereas female gender was associated with being in trajectories of more severe PTS symptoms. Ethnic/racial minority status did not emerge as a factor associated with more severe PTS symptom trajectories.

The study findings are in keeping with an increasing body of literature on PTS trajectories in youths: a low number of PTS symptoms was common, and delayed trajectories were not often observed. Lai et al found that low trajectories of PTS symptoms ranged from 37% to 79% across 8 studies of youths’ PTS symptom trajectories after disasters.^21^ Little to no evidence of a delayed trajectory was found in 5 of the 8 studies reviewed. However, those findings were limited because the systematic review approach did not allow for applying a set of consistent analyses to understand PTS symptom trajectories over time. The findings of the present study suggest that if youths do not report substantial PTS symptoms early after a disaster, they are not likely to develop substantial PTS symptoms later.

The study findings are similar to those of literature on low trajectories of PTS symptoms among adults but contrast with adult findings on delayed trajectories. A review of 57 studies of adults after trauma found that a low number of distress symptoms were observed among 65.7% of participants.^18^ However, this same review of literature on adult trauma found delayed trajectories in 8.9% of the adults studied.^18^

A small proportion of youths (10%) in this study reported a high number of chronic symptoms initially and that increased over time. This finding is consistent with studies of trauma-exposed adults, in which 10.6% of adults across studies exhibited chronic symptoms.^18^ This finding is also consistent with a recent study of 346 children (grades 4-6) followed up for 4 years after an enhanced Fujita scale (EF)-4 tornado, in which 7% of the children exhibited a chronic trajectory.^28^

In addition to the results that 23% of youths fit a recovery trajectory, this study’s findings suggest that about one-third of youths who report initially high levels of distress may develop chronic symptoms. Initial efforts to triage disaster-exposed youths based on increased PTS symptoms may identify youths who will continue to report chronic symptoms and those who will recover. Therefore, screening based on early severity of PTS symptoms alone may be insufficient for identifying those needing intensive interventions, especially when resources are limited. Additional predisaster characteristics and postdisaster recovery factors (eg, anxiety, low social support, or more life stress) may help differentiate trajectories. At a minimum, the results suggest there may be a need for ongoing monitoring of youths who initially report substantially elevated PTS symptoms. Youths with moderate symptoms also may benefit from preventive interventions.

Factors associated with more severe trajectories were examined. Older age was associated with fewer PTS symptoms, whereas female gender was associated with chronic PTS symptoms, consistent with prior research.^20,31,32,33^ These factors warrant inclusion in predictive models as researchers develop clinical tools for working with youths after a disaster. These are also factors to highlight for clinicians as they work with youths after disasters. In this study, younger children were elementary-school–aged children. Elementary schools may warrant special attention in postdisaster efforts to support children. Contrary to hypotheses, racial/ethnic minority status was not associated with more severe PTS symptom trajectories. Minority status is often a proxy for experiences of racism and systemic disparities, which should be measured explicitly in future studies.

### Limitations

This study has limitations. All the studies included were posthurricane studies. Findings may not generalize to other types of disaster events (eg, earthquakes or terrorist attacks). In addition, race/ethnicity was measured as a dichotomous variable. Additional research is needed with more diverse cohorts. We also used latent-class linear growth analysis to identify trajectories. Although this is a robust analytic approach, any single analysis approach should be interpreted with caution. Future studies examining quadratic and cubic trends are needed.^50^ In addition, a limited number of factors were examined in this study. We were not able to examine disaster exposure from each disaster given the timing of exposure assessments. Exposure is an important factor to consider in understanding resilience.^33,51,52^ Future pooled studies should incorporate additional factors such as high anxiety, low social support, poor regulation of emotions, life stress,^20^ and co-occurring depression.^39^ Future pooled analyses could be facilitated through agreement on common assessments of PTS symptoms, measurement of functional impairment, and commitment to sharing data in repositories.

## Conclusions

 In this cohort study, few youths reported chronic distress, and trajectories among most youths reflected recovery or low-decreasing PTS symptoms. Older age and male gender were factors associated with decreased odds of a chronic trajectory. These findings may guide policy makers to effectively implement stepped care models for youths after a disaster. The results also highlight the need for health surveillance systems after disasters because many youths in this study reported elevated PTS symptoms.

## References

[1] Save the Children. Rewriting the future for children: annual report 2007. Published 2008 Accessed July 23, 2020. https://www.savethechildren.org/content/dam/usa/reports/annual-report/annual-report/sc-2007-annualreport.pdf

[2] United Nations Office for Disaster Risk Reduction Comprehensive school safety. Published 2017 Accessed July 23, 2020. https://www.preventionweb.net/files/51335_cssbooklet2017updated.pdf

[3] La Greca AM, Lai BS, Joormann J, Auslander BB, Short MA Children’s risk and resilience following a natural disaster: genetic vulnerability, posttraumatic stress, and depression. J Affect Disord. 2013;151(3):860-867. doi:10.1016/j.jad.2013.07.024 24035489

[4] Lai BS, Kelley ML, Harrison KM, Thompson JE, Self-Brown S Posttraumatic stress, anxiety, and depression symptoms among children after Hurricane Katrina: a latent profile analysis. J Child Fam Stud. 2015;24(5):1262-1270. doi:10.1007/s10826-014-9934-3 25892902 PMC4399827

[5] Weems CF, Taylor LK, Cannon MF, Post traumatic stress, context, and the lingering effects of the Hurricane Katrina disaster among ethnic minority youth. J Abnorm Child Psychol. 2010;38(1):49-56. doi:10.1007/s10802-009-9352-y 19707864

[6] Roussos A, Goenjian AK, Steinberg AM, Posttraumatic stress and depressive reactions among children and adolescents after the 1999 earthquake in Ano Liosia, Greece. Am J Psychiatry. 2005;162(3):530-537. doi:10.1176/appi.ajp.162.3.530 15741470

[7] Farrugia PL, Mills KL, Barrett E, Childhood trauma among individuals with co-morbid substance use and post traumatic stress disorder. Ment Health Subst Use. 2011;4(4):314-326. doi:10.1080/17523281.2011.598462 21984884 PMC3188414

[8] Furr JM, Comer JS, Edmunds JM, Kendall PC Disasters and youth: a meta-analytic examination of posttraumatic stress. J Consult Clin Psychol. 2010;78(6):765-780. doi:10.1037/a0021482 21114340

[9] Hadi F, Lai BS, Llabre MM Life outcomes influenced by war-related experiences during the Gulf crisis. Anxiety Stress Coping. 2014;27(2):156-175. doi:10.1080/10615806.2013.832219 24003829 PMC3877742

[10] Lai BS, La Greca AM, Llabre MM Children’s sedentary activity after hurricane exposure. Psychol Trauma. 2014;6(3):280-289. doi:10.1037/a0033331

[11] Schonhardt S Paving a path to recovery: some issues worth watching as the Philippines moves down the road to recovery after Typhoon Haiyan. *The Wall Street Journal* Published May 20, 2013. Accessed January 3, 2021. http://online.wsj.com/news/articles/SB10001424052702303653004579211483596866854

[12] Pfefferbaum B, North CS Child disaster mental health services: a review of the system of care, assessment approaches, and evidence base for intervention. Curr Psychiatry Rep. 2016;18(1):5. doi:10.1007/s11920-015-0647-0 26719308

[13] Ronan KR, Johnston DM Promoting Community Resilience in Disasters: The Role for Schools, Youth, and Families. Springer Science & Business Media; 2005. doi:10.1007/b102725

[14] Garrett AL, Grant R, Madrid P, Brito A, Abramson D, Redlener I Children and megadisasters: lessons learned in the new millennium. Adv Pediatr. 2007;54(1):189-214. doi:10.1016/j.yapd.2007.03.011 17918472

[15] Institute of Medicine In: Wizemann T, Reeve M, Altevogt BM, eds. Preparedness, Response, and Recovery Considerations for Children and Families: Workshop Summary. The National Academies Press; 2014. 24354032

[16] Kessler RC, Keane TM, Ursano RJ, Mokdad A, Zaslavsky AM Sample and design considerations in post-disaster mental health needs assessment tracking surveys. Int J Methods Psychiatr Res. 2008;17(S2)(suppl 2):S6-S20. doi:10.1002/mpr.269 19035440 PMC3081100

[17] Pfefferbaum B, Weems CF, Scott BG, Research methods in child disaster studies: a review of studies generated by the September 11, 2001, terrorist attacks; the 2004 Indian Ocean tsunami; and Hurricane Katrina. Child Youth Care Forum. 2013;42(4):285-337. doi:10.1007/s10566-013-9211-4 24443635 PMC3892998

[18] Galatzer-Levy IR, Huang SH, Bonanno GA Trajectories of resilience and dysfunction following potential trauma: a review and statistical evaluation. Clin Psychol Rev. 2018;63:41-55. doi:10.1016/j.cpr.2018.05.008 29902711

[19] Lowe SR, Rhodes JE Trajectories of psychological distress among low-income, female survivors of Hurricane Katrina. Am J Orthopsychiatry. 2013;83(2 Pt 3):398-412. doi:10.1111/ajop.12019 23889030 PMC3999519

[20] La Greca AM, Lai BS, Llabre MM, Silverman WK, Vernberg EM, Prinstein MJ Children’s postdisaster trajectories of PTS symptoms: predicting chronic distress. Child Youth Care Forum. 2013;42(4):351-369. doi:10.1007/s10566-013-9206-1 24683300 PMC3964678

[21] Lai BS, Lewis R, Livings MS, La Greca AM, Esnard A-M Posttraumatic stress symptom trajectories among children after disaster exposure: a review. J Trauma Stress. 2017;30(6):571-582. doi:10.1002/jts.22242 29193316 PMC5953201

[22] Bonanno GA, Brewin CR, Kaniasty K, Greca AM Weighing the costs of disaster: consequences, risks, and resilience in individuals, families, and communities. Psychol Sci Public Interest. 2010;11(1):1-49. doi:10.1177/1529100610387086 26168411

[23] Bonanno GA, Mancini AD The human capacity to thrive in the face of potential trauma. Pediatrics. 2008;121(2):369-375. doi:10.1542/peds.2007-1648 18245429

[24] Anderson WA Women and children facing disaster. In: Kreimer A, Arnold M, eds. Managing Disaster Risk in Emerging Economies. The World Bank; 2000:99-105.

[25] Fothergill A, Peek LA Children of Katrina. University of Texas Press; 2015.

[26] Peek L. Children and disasters: understanding vulnerability, developing capacities, and promoting resilience—an introduction. Child Youth Environ. 2008;18(1):1-29.

[27] De Young AC, Kenardy JA, Cobham VE, Kimble R Prevalence, comorbidity and course of trauma reactions in young burn-injured children. J Child Psychol Psychiatry. 2012;53(1):56-63. doi:10.1111/j.1469-7610.2011.02431.x 21671940

[28] McDonald KL, Vernberg EM, Lochman JE, Trajectories of tornado-related posttraumatic stress symptoms and pre-exposure predictors in a sample of at-risk youth. J Consult Clin Psychol. 2019;87(11):1003-1018. doi:10.1037/ccp0000432 31556648 PMC6800789

[29] Sigurdardottir S, Andelic N, Roe C, Schanke AK Identifying longitudinal trajectories of emotional distress symptoms 5 years after traumatic brain injury. Brain Inj. 2014;28(12):1542-1550. doi:10.3109/02699052.2014.934285 25029224

[30] US Global Change Research Program The Impacts of Climate Change on Human Health in the United States: A Scientific Assessment. US Global Change Research Program; 2016.

[31] Fan F, Long K, Zhou Y, Zheng Y, Liu X Longitudinal trajectories of post-traumatic stress disorder symptoms among adolescents after the Wenchuan earthquake in China. Psychol Med. 2015;45(13):2885-2896. doi:10.1017/S0033291715000884 25990926

[32] Kronenberg ME, Hansel TC, Brennan AM, Osofsky HJ, Osofsky JD, Lawrason B Children of Katrina: lessons learned about postdisaster symptoms and recovery patterns. Child Dev. 2010;81(4):1241-1259. doi:10.1111/j.1467-8624.2010.01465.x 20636693

[33] Osofsky JD, Osofsky HJ, Weems CF, King LS, Hansel TC Trajectories of post-traumatic stress disorder symptoms among youth exposed to both natural and technological disasters. J Child Psychol Psychiatry. 2015;56(12):1347-1355. doi:10.1111/jcpp.12420 25898776

[34] Self-Brown S, Lai BS, Thompson JE, McGill T, Kelley ML Posttraumatic stress disorder symptom trajectories in Hurricane Katrina affected youth. J Affect Disord. 2013;147(1-3):198-204. doi:10.1016/j.jad.2012.11.002 23206321 PMC4231137

[35] Cutter SL Hazards, Vulnerability and Environmental Justice. Routledge; 2012.

[36] Tierney K. The Social Roots of Risk: Producing Disasters, Promoting Resilience. Stanford University Press; 2014.

[37] La Greca AM, Silverman WK, Lai B, Jaccard J Hurricane-related exposure experiences and stressors, other life events, and social support: concurrent and prospective impact on children’s persistent posttraumatic stress symptoms. J Consult Clin Psychol. 2010;78(6):794-805. doi:10.1037/a0020775 20939624

[38] La Greca A, Silverman WK, Vernberg EM, Prinstein MJ Symptoms of posttraumatic stress in children after Hurricane Andrew: a prospective study. J Consult Clin Psychol. 1996;64(4):712-723. doi:10.1037/0022-006X.64.4.712 8803361

[39] Lai BS, La Greca AM, Auslander BA, Short MB Children’s symptoms of posttraumatic stress and depression after a natural disaster: comorbidity and risk factors. J Affect Disord. 2013;146(1):71-78. doi:10.1016/j.jad.2012.08.041 22974469 PMC3640419

[40] Pynoos RS, Rodriguez N, Steinberg A, Stuber M, Frederick C The UCLA PTSD Reaction Index for DSM IV (Revision 1). UCLA Trauma Psychiatry Program; 1998.

[41] Steinberg AM, Brymer MJ, Decker KB, Pynoos RS The University of California at Los Angeles post-traumatic stress disorder reaction index. Curr Psychiatry Rep. 2004;6(2):96-100. doi:10.1007/s11920-004-0048-2 15038911

[42] Pynoos RS, Frederick C, Nader K, Life threat and posttraumatic stress in school-age children. Arch Gen Psychiatry. 1987;44(12):1057-1063. doi:10.1001/archpsyc.1987.01800240031005 3689093

[43] Vernberg EM, Silverman WK, La Greca AM, Prinstein MJ Prediction of posttraumatic stress symptoms in children after hurricane Andrew. J Abnorm Psychol. 1996;105(2):237-248. doi:10.1037/0021-843X.105.2.237 8723005

[44] Costa NM, Weems CF, Pina AA Hurricane Katrina and youth anxiety: the role of perceived attachment beliefs and parenting behaviors. J Anxiety Disord. 2009;23(7):935-941. doi:10.1016/j.janxdis.2009.06.002 19577899

[45] *SAS/ACCESS* Version 9.4. SAS Institute Inc; 2013. Accessed January 7, 2021. https://www.sas.com/en_us/software/access.html

[46] Muthén LK, Muthén BO *Mplus User’s Guide* 6th ed. Muthén & Muthén; 1998.

[47] Berlin KS, Williams NA, Parra GR An introduction to latent variable mixture modeling (part 1): overview and cross-sectional latent class and latent profile analyses. J Pediatr Psychol. 2014;39(2):174-187. doi:10.1093/jpepsy/jst084 24277769

[48] Nylund KL, Asparouhov T, Muthén BO Deciding on the number of classes in latent class analysis and growth mixture modeling: a Monte Carlo simulation study. *Struct Equ Modeling* 2007;14(4):535-569.

[49] Asparouhov T, Muthen B Auxiliary variables in mixture modeling: a 3-step approach using Mplus. Published 2013. Accessed July 23, 2020. https://www.statmodel.com/examples/webnotes/AuxMixture_submitted_corrected_webnote.pdf

[50] Twisk J, Hoekstra T Classifying developmental trajectories over time should be done with great caution: a comparison between methods. J Clin Epidemiol. 2012;65(10):1078-1087. doi:10.1016/j.jclinepi.2012.04.010 22818946

[51] Weems CF, Graham RA Resilience and trajectories of posttraumatic stress among youth exposed to disaster. J Child Adolesc Psychopharmacol. 2014;24(1):2-8. doi:10.1089/cap.2013.0042 24200122

[52] Infurna FJ, Luthar SS Resilience has been and will always be, but rates declared are inevitably suspect: reply to Galatzer-Levy and Bonanno (2016). Perspect Psychol Sci. 2016;11(2):199-201. doi:10.1177/1745691615621281 26993274 PMC4806402

